# Pickle water ameliorates castor oil-induced diarrhea in mice by regulating the homeostasis of the gut microbiota and intestinal mucosal barrier

**DOI:** 10.3389/fnut.2024.1455091

**Published:** 2024-09-12

**Authors:** Tian Zhou, Dongmei Long, Maoting Zhou, Xianghong Hu, Yu Wang, Xing Wang

**Affiliations:** ^1^The Affiliated Children's Hospital of Zhengzhou University, Zhengzhou, China; ^2^Nanchong Key Laboratory of Disease Prevention, Control and Detection in Livestock and Poultry, Nanchong Vocational and Technical College, Nanchong, China; ^3^School of Pharmacy, North Sichuan Medical College, Nanchong, China

**Keywords:** pickle water, oxidative stress, 16S rRNA, intestinal flora, diarrhea

## Abstract

**Introduction:**

Diarrhea is a common clinical condition that can potentially be fatal. Current treatment options often have side effects, such as constipation and vomiting, and there remains a need for more effective therapies. Pickled vegetables, a famous traditional food in China, have been suggested in clinical studies to alleviate diarrhea in children, particularly through the use of pickle water (PW). However, the pharmacological effects and mechanisms of PW on intestinal health remain unclear. This study aimed to explore the protective effects of PW on castor oil-induced diarrhea in ICR mice and to investigate its potential mechanisms.

**Methods:**

To evaluate the antidiarrheal effects of PW, we used a castor oil-induced diarrhea model in ICR mice. Various indices were measured to assess the severity of diarrhea. After euthanizing the mice, oxidative stress markers in the ileum were assessed using biochemical methods, and the expression of tight junction-related proteins in the ileum was analyzed using Western blot. Additionally, 16S rRNA high-throughput sequencing was used to evaluate the diversity and composition of the intestinal flora.

**Results:**

The results showed that PW supplementation reduced body weight without significantly affecting organ index and liver function in the castor oil-induced diarrhea mice. PW also effectively reduced the dilution rate, diarrhea index, average loose stool grade, propelling distance of carbon powder, and intestinal propulsive rate while improving the pathological abnormality in the ileum. Furthermore, PW enhanced the activities of total antioxidant capacity (T-AOC), glutathione peroxidase (GSH-PX), and catalase (CAT) while reducing malonaldehyde (MDA) levels. PW also increased the expression of tight junction proteins zonula occludens-1 (ZO-1) and occludin in the ileum. Additionally, the analysis of 16S rDNA revealed that PW increased both α and β diversity, improved the composition of the intestinal flora, and restored it to a normal level.

**Discussion:**

Collectively, dietary PW administration ameliorates Castor oil-induced diarrhea by restoring tight junctions between intestinal mucosal cells, suppressing oxidative stress, and regulating the composition of intestinal flora. These findings suggest that PW may be a promising strategy for managing diarrhea.

## 1 Introduction

Diarrhea is a common intestinal disease with high incidence, caused by changes in electrolyte absorption/secretion or the accumulation of non-absorbable/osmotically active substances in the intestinal cavity (1). Intestinal infections, abnormal intestinal contractions, imbalances in intestinal flora, poor food tolerance, or stress can lead to diarrhea. The pathological basis of diarrhea includes intestinal edema, mucosal inflammation, increased intestinal secretion, and motility (2). Diarrhea is a leading cause of morbidity and mortality due to global infectious disease epidemics among infants, young children, and immunocompromised individuals, resulting in more than 500,000 child deaths annually (3, 4). Prolonged diarrhea in children can lead to malnutrition and growth retardation. It can also impact the immune system (5).

Moreover, chronic diarrhea significantly affects patients' studies, work, and social activities, reduces their quality of life, and further impacts mental health, creating economic and medical burdens for patients, families, and society. Currently, clinical treatment and relief of diarrhea mainly rely on medications (such as anticholinergic drugs, selective serotonin reuptake inhibitors, bile acid binders, tricyclic antidepressants, and somatostatin analogs) and non-drug therapies (diet regulation, psychotherapy, and exercise therapy) (4, 6). However, the indications for these drugs are limited, and they are often accompanied by adverse effects. For instance, abuse of antibiotics leads to bacterial resistance, intestinal flora imbalance, and secondary fungal enteritis (7). Most drugs are symptomatic treatments, and the treatment cycle is short. Patients are prone to relapse due to factors such as diet and mood, and the treatment effect is not ideal, which affects the quality of patients' daily lives and brings unnecessary economic burdens to patients (8). Therefore, developing effective drugs or strategies for treating diarrhea is necessary.

The pathogenesis of diarrhea involves various aspects, including intestinal infections leading to inflammatory reactions, apoptosis, gastrointestinal hormone secretion disorders, intestinal mucosal injury, and water and electrolyte secretion disorders (9, 10). Among these, an imbalance in intestinal microflora is recognized as a significant factor that increases sensitivity to various pathogens and promotes the occurrence of diarrhea (11). Under normal conditions, bacteria, fungi, protozoa, and viruses in the intestines maintain a dynamic balance, participating in nutrient absorption and physiological functions and differing microbial compositions in various parts, thus playing different roles in maintaining normal functions of the intestines (12). However, changes in the composition and diversity of intestinal microorganisms caused by diet, drugs, pathogens, and environmental factors can affect health (13). For example, *Escherichia coli*, Shigella, Salmonella, Campylobacter, *Clostridium difficile*, and Aeromonas can all cause diarrhea (9). Studies have shown that probiotics can effectively prevent and treat diarrhea (13). Therefore, regulating intestinal flora homeostasis and restoring its structure is essential for preventing and treating diarrhea.

Pickled vegetables are foods made by fermenting fresh vegetables with seasonings, and they have a history of over 3,000 years in China. They are popular among Chinese people due to their simple production and delicious taste. Sichuan Kimchi is one of the most famous pickled vegetables in China, and its production process involves the fermentation of lactic acid bacteria to produce high concentrations of lactic acid, resulting in a delicious taste after long-term storage (14, 15). Moreover, it was found that the production process of Sichuan pickled vegetables involves the reuse of salt solution, so long-term and repeated fermentation produced a complex microbial system containing rich bacterial flora, including known strains such as *Lactobacillus brevis, Lactobacillus plantarum, Pediococcus* ethanol-resistant, *Lactobacillus casei, Lactobacillus pentosaceus, Lactobacillus sakai, Lactobacillus foodborne*, and *Leuconostoc mesenteroides* (16). Pickles also contain nutrients such as lactic acid, vitamins, and amino acids. Intestinal flora imbalance is one of the main causes of diarrhea, and studies have shown that pickle water (PW) can relieve diarrhea in children (15). However, there have been few reports, both domestically and internationally, on the role of pickle water in improving diarrhea and regulating intestinal microbial structure. Therefore, this study aimed to explore the protective effect of pickle water on diarrhea caused by castor oil and further investigate its possible mechanism from the perspective of intestinal flora.

## 2 Materials and methods

### 2.1 Reagents and antibodies

Pickle juice was collected from pickled vegetables in Leshan City, Sichuan Province. Loperamide hydrochloride was bought from Yuanye Biotechnology Co., Ltd. (Shanghai, China). Kits for measuring tumor necrosis factor-alpha (TNF-α) and interleukin-6 (IL-6) contents were from Beijing Dongge Boye Biotechnology Co. Ltd. (Beijing, China). The kits of alanine aminotransferase (ALT), aspartate aminotransferase (AST), total antioxidant capacity (T-AOC), glutathione peroxidase (GSH-PX), catalase (CAT), and malonaldehyde (MDA) were acquired from Nanjing Jiancheng Bioengineering Institute (Nanjing, Jiangsu, China). The activated carbon was purchased from National Medicine Group Chemical Reagent Co., Ltd. (Shanghai, China). Antibodies against zonula occludens-1 (ZO-1) and occludin were purchased from Cell Signaling Technology (Danvers, MA, USA). The secondary antibody, RIPA lysis buffer, enhanced chemiluminescence (ECL) reagent, protease and phosphatase inhibitors, and bicinchoninic acid (BCA) protein kit were acquired from Applygen Technologies (Beijing, China).

### 2.2 Animals

Fifty male ICR mice (6 weeks old, weighing 20 ± 2 g) were purchased from HFK Bio-Technology. Co., Ltd. (Beijing, China); the company's license number is SCXK (Jing) (2019−0008). The mice had free access to food and water and were housed under a 12-h light/dark cycle (humidity, 55 ± 10%; temperature, 23 ± 2°C) throughout the study. All experimental procedures were conducted in accordance with the Guidelines for the Care and Use of Laboratory Animals (GB14925-2001 and MOST 2006a) and approved by the Experimental Animal Welfare Ethics Committee of North Sichuan Medical College.

### 2.3 Experimental design

After 1 week of adaptive feeding, the ICR mice were randomly divided into the following groups based on their weight: (1) normal control group (Nor), (2) diarrhea model group (Mod), (3) positive control group (Loperamide hydrochloride, Lop, 10 mL/kg), (4) low dose group of pickle water (PW-L, 5 mL/kg), and (5) high dose group of pickle water (PW-H, 10 mL/kg). The Lop, PW-L, and PW-H groups received the corresponding treatments by gavage, while the Nor and Mod groups were given the same volumes of water. After 10 days of continuous administration, the mice in the administration and Mod groups were given castor oil (20 mL/kg) at 2:00 p.m. for five consecutive days to induce diarrhea. During the experiment, the mice's body weight, food intake, and water consumption were measured daily. On the second day after the last administration of castor oil, the mice were fasted for 12 h, and the mice were euthanized after carrying out a small intestine propulsion experiment. Serum was collected when the mice were euthanized, and the liver, kidney, heart, and spleen were taken and weighed. The small intestine was rinsed with normal saline; one portion was fixed in 4% paraformaldehyde for pathological staining, and the remainder was placed in an EP tube and frozen at −80°C for biochemical index determination. The organ index was calculated using the formula: organ index = organ weight (mg)/body weight (g).

### 2.4 Small intestine propulsion experiment

On the second day after the last administration of castor oil, the mice were made to fast for 12 h and orally administered 0.5% activated carbon (10 mL/kg). After 30 min, the mice were anesthetized with 10% chloral hydrate (10 mL/kg) and euthanized. The abdomen of each mouse was opened, and the intestine was quickly removed and placed in a clean tray. The distance from the pylorus to the ileocecal valve was measured as the total length, and the distance from the pylorus to the end of the charcoal powder was measured as the propulsion length. The intestinal propulsion rate was calculated using the following formula: intestinal propulsive rate = propulsion length/total length.

### 2.5 Diarrhea indices determination

After the last gavage of castor oil and PW, the mice were placed in clean observation cages covered with filter paper, which was changed every h for 6 h. Diarrhea was evaluated by recording the number of defecations, the number of loose stools, the loose stools grade, and the diarrhea index. The loose stools grade indicated the severity of loose stools and was divided into four grades based on the diameter (cm) of the polluted area on the filter paper: Grade I (< 1 cm), Grade II (1-1.9 cm), Grade III (2–3 cm), and Grade IV (>3 cm). In statistics, the series of loose stools was measured individually, and the total was divided by the number of loose stools to obtain the average loose stool grade. The average loose stool grade was calculated using the formula: average grade of loose stool = total loose stool grade/number of loose stools. The loose stool rate was calculated using the formula: loose stools rate = the number of loose stools in mice/total defecation times of mice × 100%. The diarrhea score was calculated as follows: diarrhea index = diarrhea stool rate × degree of diarrhea.

### 2.6 Analysis of biochemical indexes in the serum and ileum

Blood samples were obtained from the mouse orbit, kept at room temperature for 2 h, and centrifuged at 4°C and 5,000 rpm for 10 min, with the supernatant collected in a clean EP tube. The activities of ALT and AST were determined according to the kit instructions. A certain weight of small intestine tissue was accurately weighed for the ileum, and physiological saline was added in a weight-to-volume ratio of 1:9. The tissue was then cut and homogenized using a mechanical homogenizer. After standing on ice for 40 min, the samples were centrifuged at 4°C and 12,000 rpm for 10 min, and the supernatant was collected in a new EP tube. The T-AOC, GSH-PX, CAT, and MDA levels in the supernatant were determined according to the instructions, and the results were normalized to protein concentration.

### 2.7 Histopathological analysis

The small intestines were fixed in 4% paraformaldehyde for 24 h. They were then embedded in paraffin and cut into 5 μm thick slices using a microtome. According to standard protocols, the slices were stained with hematoxylin and eosin (H&E). Finally, they were observed and photographed using a microscope (NIKON ECLIPSE E100, Nikon, Japan) to evaluate the pathological changes in the small intestine. According to the method described previously, Chiu's method was used to semi-quantitatively score the changes in intestinal villi and glands. The selection of areas scoring was blindly analyzed by people (17, 18).

### 2.8 Western blotting analysis

To extract protein from small intestine tissue, a specified amount of tissue was accurately weighed, and RIPA lysis buffer containing phenylmethylsulfonyl fluoride (PMSF) was added in a weight-to-volume ratio of 1:9. The tissues were then cut and homogenized using a mechanical homogenizer and left to stand on ice for 40 min. The samples were centrifuged at 4°C and 12,000 rpm for 10 min to separate the supernatant. The protein concentration of the supernatant was determined using the BCA method, and all samples were adjusted to the same concentration. Next, 5 × loading buffer was added to the samples, which were then boiled at 100°C for 10 min. Sodium dodecyl sulfate-polyacrylamide gel electrophoresis (SDS-PAGE) and subsequent data analysis were performed as previously described (19).

### 2.9 Fecal pellet collection and 16S rDNA sequencing

After the last administration, the mice were placed in sterilized observation cages, and 200 mg of feces from each mouse were collected and stored in clean EP tubes, which were then frozen in liquid nitrogen. The composition and structure of the intestinal flora were examined using 16S rRNA gene sequencing by Shanghai Biotree Biotech Co., Ltd. (Shanghai, China) with advanced technology. The QIAamp Fast DNA Stool Mini Kit (Qiagen, CA, United States) was used according to the manufacturer's instructions to extract and purify DNA from mouse feces. The quantity and concentration of DNA were measured using gel electrophoresis. The hypervariable region V3-V4 of the bacterial 16S rRNA gene was amplified using standard primer pairs and then purified and quantified. The 16S rDNA PCR products were sequenced on an Illumina MiSeq (PE300), and the data were analyzed accordingly. Using QIIME2 software, α-diversity (Chao1, Shannon, and Simpson indices) and β-diversity were analyzed. Using R software, principal component analysis (PCA), principal coordinate analysis (PCoA), and non-metric multidimensional scaling (NMDS), dimensionality reduction plots were created. The amplified sequences were merged, and operational taxonomic units (OTUs) were defined based on a sequence similarity threshold of 97%. Bar plots and PCoA plots were generated using R software. The Mann–Whitney *U*-test and the Kruskal–Wallis test were used for comparative analysis.

### 2.10 Statistical analysis

The Shapiro–Wilk method in GraphPad Prism 6.0 (GraphPad Software Inc.) was used to test the normal distribution of all the results, and GraphPad Prism 6.0 was used to generate figures and analyze data. All data are expressed as mean ± standard error of the mean (SEM). Differences between the two groups were analyzed by using a two-tailed Student's *t*-test, and the differences between multiple groups were analyzed using a one-way analysis of variance (ANOVA), followed by Bonferroni's correction. Correlation analysis was carried out using Pearson's correlation analysis. Differences of *P* < 0.05 were considered statistically significant.

## 3 Results

### 3.1 Effects of PW on basic metabolic index and organ index in mice with castor oil-induced diarrhea

**Figure 1:**
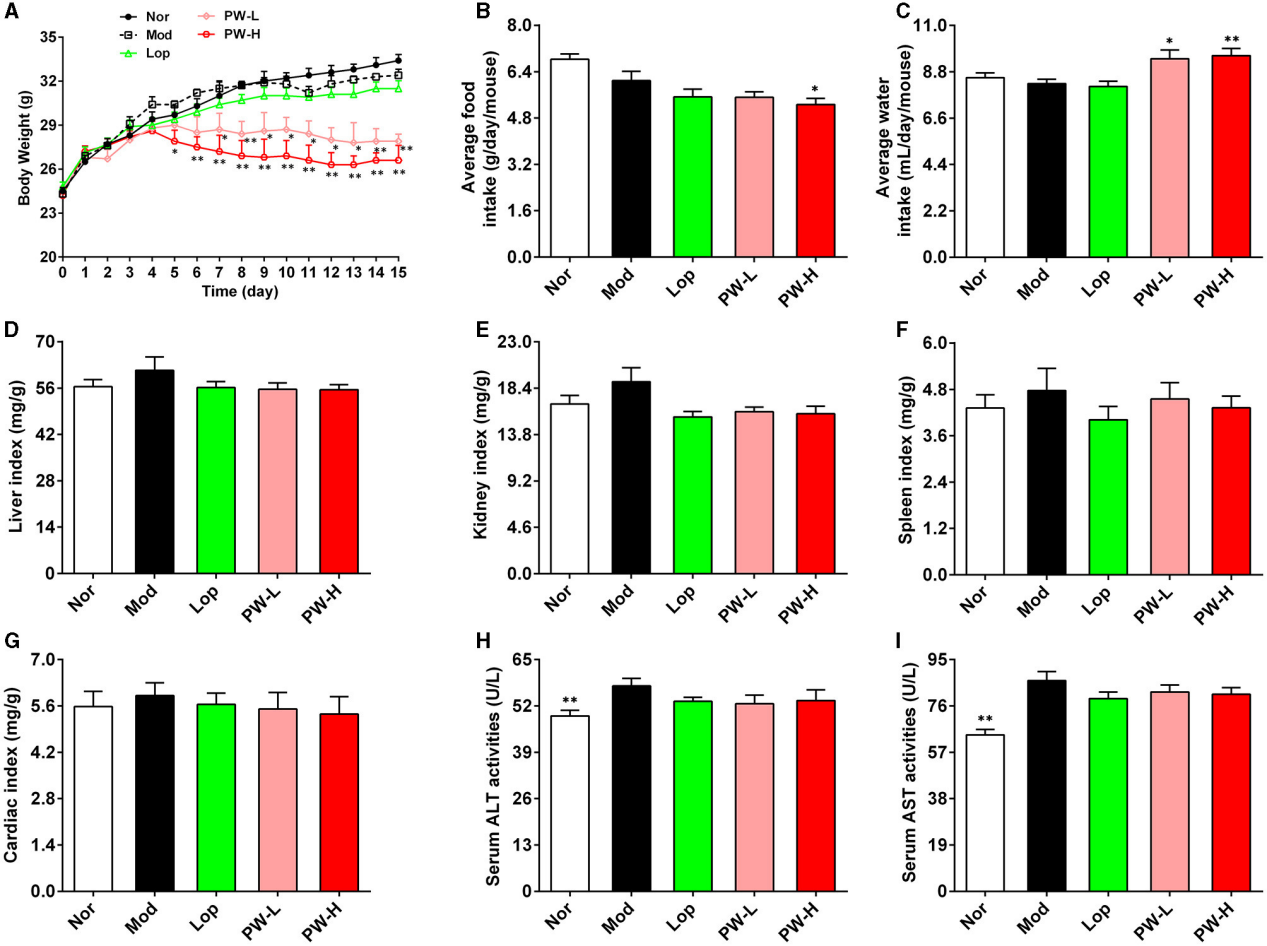
Effects of PW on basic metabolic index and organ index in mice with castor oil-induced diarrhea (*n* = 10, means ± SEM). **(A)** Body weight. **(B)** Average food intake. **(C)** Average water consumption. **(D)** Liver index. **(E)** Kidney index. **(F)** Spleen index. **(G)** Cardiac index. **(H)** Serum ALT activities. **(I)** Serum AST activities. *P < 0.05; **P < 0.01 vs. the Mod group.

Relative to the Nor group, the Mod group mice showed listlessness, rough hair, loss of appetite, and slow movement, and the feces were thin, soft, shapeless, and muddy, and some even showed watery stools, but the PW improved the stool characteristics and mental state of mice. Moreover, we investigated the effect of PW on the basic metabolic indexes of the mice. As shown in Figures 1A–C, the body weight of mice in the Nor, Mod, and Lop groups increased steadily throughout the experiment, with no significant differences among the three groups. The mice treated with PW exhibited a notable, dose-dependent decrease in body weight compared to the Mod group. Regarding food intake and water consumption, there were no significant differences among the groups Nor, Mod, and Lop. However, mice in the PW-L and PW-H groups showed higher levels of water consumption than those in the Mod group, while the PW-H group had lower food intake compared to the Mod group. The results of the organ index are shown in Figures 1D–G. Compared to the Nor group, the liver index, kidney index, spleen index, and heart index in the Mod group showed an increasing trend, whereas the organ indices in the other groups decreased compared to the Mod group. However, these differences were not statistically significant. The results of Figures 1H, I showed that, in contrast with Nor group mice, ALT and AST activities were significantly higher in Mod group mice. Moreover, Lop and PW tended to decrease the activities of ALT and AST in the serum of mice.

### 3.2 Effects of PW on castor oil-induced diarrhea in mice

To investigate the potential of PW in improving diarrhea induced by castor oil in mice, the relevant indices of diarrhea were evaluated. As displayed in Table 1, compared to the Nor group, the mice in the Mod group exhibited a higher diarrhea stool rate, average loose stool grade, and diarrhea index, suggesting the diarrhea model has been successfully formed. Nevertheless, compared to the Mod group, these three indexes for evaluating diarrhea decreased significantly after 15 days of administration of PW-H. Lop or PW-L treatment remarkably reduced the diarrhea stool rate diarrhea index and showed a tendency to decrease the average loose stool grade. Cumulatively, these data indicate that PW can effectively improve diarrhea induced by castor oil.

**Table 1:** Effects of PW on castor oil-induced diarrhea in mice.

**Group**	**Dose (mL/kg)**	**Diarrhea stool rate (%)**	**Average loose stool grade**	**Diarrhea index**
Nor	–	–	–	–
Mod	–	68.9 ± 9.6	2.21 ± 0.57	1.49 ± 0.26
Lop	20	32.7 ± 9.7^**^	2.01 ± 0.56	0.64 ± 0.19^**^
PW-L	10	53.5 ± 12.8^*^	2.04 ± 0.53	1.04 ± 0.16^**^
PW-H	20	40.2 ± 10.6^**^	1.57 ± 0.51^*^	0.60 ± 0.19^**^

Data are presented as mean ± SEM, *n* = 10. ^*^P < 0.05; ^**^P < 0.01 vs. the Mod group.

### 3.3 Effects of PW on small intestine propulsion in mice with castor oil-induced diarrhea

To further investigate the effectiveness of PW in improving castor oil-induced diarrhea, we conducted a small intestine propulsion experiment following the euthanasia of the mice. As shown in Table 2, the propelling distance of carbon powder and the intestinal propulsive rate were 10.8 cm and 18.5% higher in the Mod groups compared to the Nor group. Conversely, the propelling distance of carbon powder and intestinal propulsive rate in the Lop, PW-L, and PW-H groups decreased significantly after 15 days of Lop and PW administration compared to the Mod group. These findings provide evidence that PW effectively alleviates castor oil-induced diarrhea.

**Table 2:** Effects of PW on small intestine propulsion in mice with castor oil-induced diarrhea.

**Group**	**Dose (mL/kg)**	**Propelling distance of carbon powder (cm)**	**Intestinal propulsive rate (%)**
Nor	–	30.4 ± 2.5^**^	60.9 ± 7.4^**^
Mod	–	41.2 ± 6.7	79.4 ± 12.8
Lop	20	29.1 ± 2.7^**^	54.4 ± 3.8^**^
PW-L	10	32.1 ± 3.1^**^	63.1 ± 6.1^*^
PW-H	20	29.0 ± 2.1^**^	56.7 ± 5.7^**^

Data are presented as mean ± SEM, *n* = 10. ^*^P < 0.05; ^**^P < 0.01 vs. the Mod group.

### 3.4 Effects of PW on intestinal morphology and intestinal integrity in mice with castor oil-induced diarrhea

**Figure 2:**
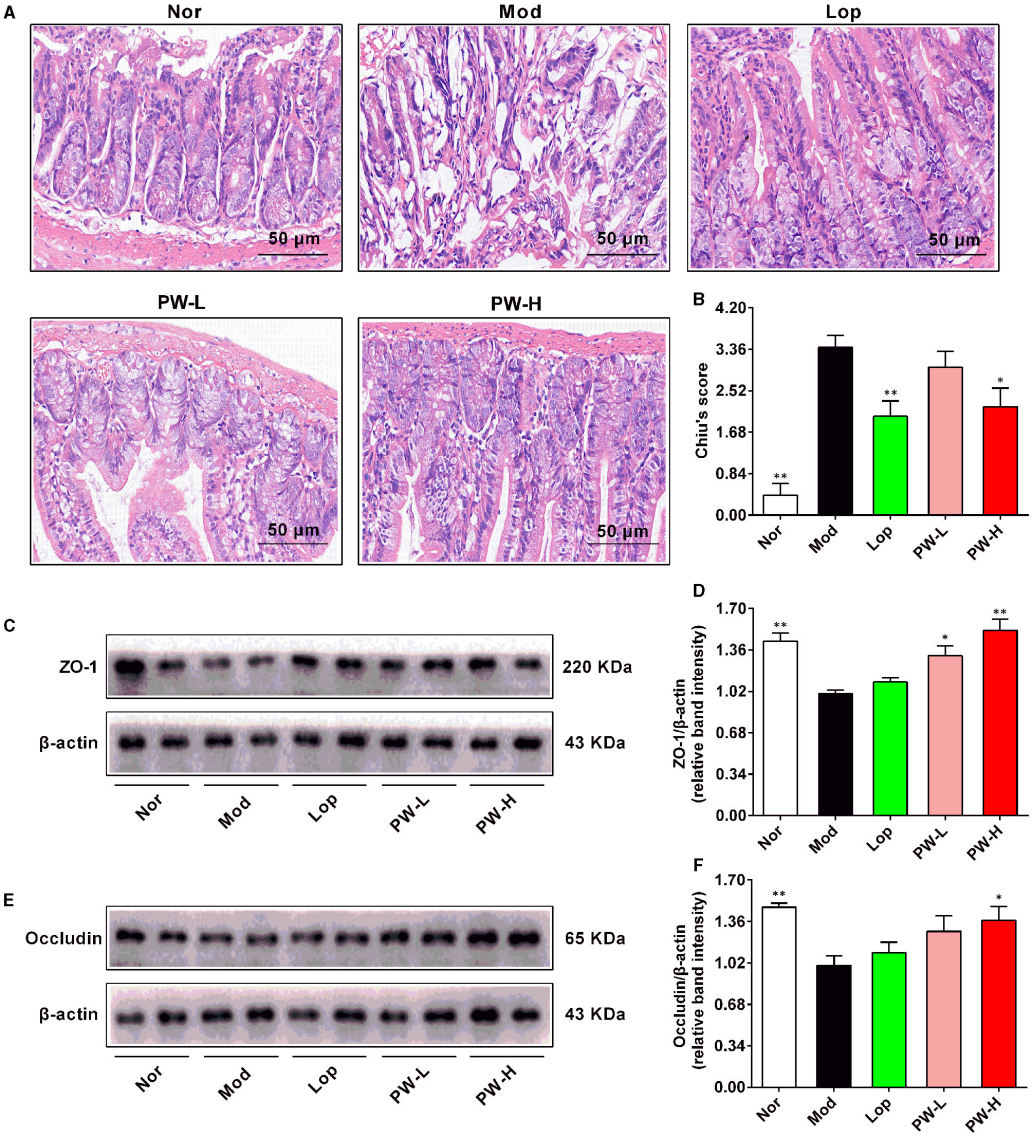
Effects of PW on intestinal morphology and intestinal integrity in mice with castor oil-induced diarrhea (*n* = 4, means ± SEM). **(A)** Representative pictures of HE staining. **(B)** Chiu's score. **(C)** The representative images of the western blot of ZO-1. **(D)** The relative density analysis of ZO-1. **(E)** The representative images of the western blot of occludin. **(F)** The relative density analysis of occludin. *P < 0.05; **P < 0.01 vs. the Mod group.

Then, the ileum of the mice was stained with H&E, and the intestinal integrity-related proteins in the ileum were determined. As demonstrated in Figure 2A, H&E staining of ileum in the Nor group exhibited normal morphology of intestinal villi, with increased numbers of immune cells such as lymphocytes and plasma cells in the lamina propria of villi and intraepithelial lymphocytes. Conversely, the ileum of the Mod group displayed epithelial swelling, villous epithelial degeneration, villous collapse, and chronic inflammatory cell infiltration. Interestingly, these abnormal changes were partially reversed after 15 days of Lop or PW treatment. The results of Chiu's score are shown in Figure 2B. Compared to the Nor group, the mice in the Mod group exhibited a higher Chiu's score.

Nevertheless, relative to the Mod group, Lop and PW-H markedly reduced Chiu's score, and PW-L tended to decrease Chiu's score, which was in agreement with the changes in the structure of the small intestine. The results of the western blot are shown in Figures 2C–F. The protein expressions of ZO-1 and occludin in the ileum of the Mod were markedly lower than those in the Nor group. However, after 15 days of Lop or PW treatment, the expression levels of these two proteins increased compared to the Mod group. Taken together, these results indicate that PW can ameliorate castor oil-induced intestinal morphology abnormalities and help maintain the integrity of the intestinal barrier.

### 3.5 Effect of PW on oxidative stress and inflammatory reaction in mice with castor oil-induced diarrhea

**Figure 3:**
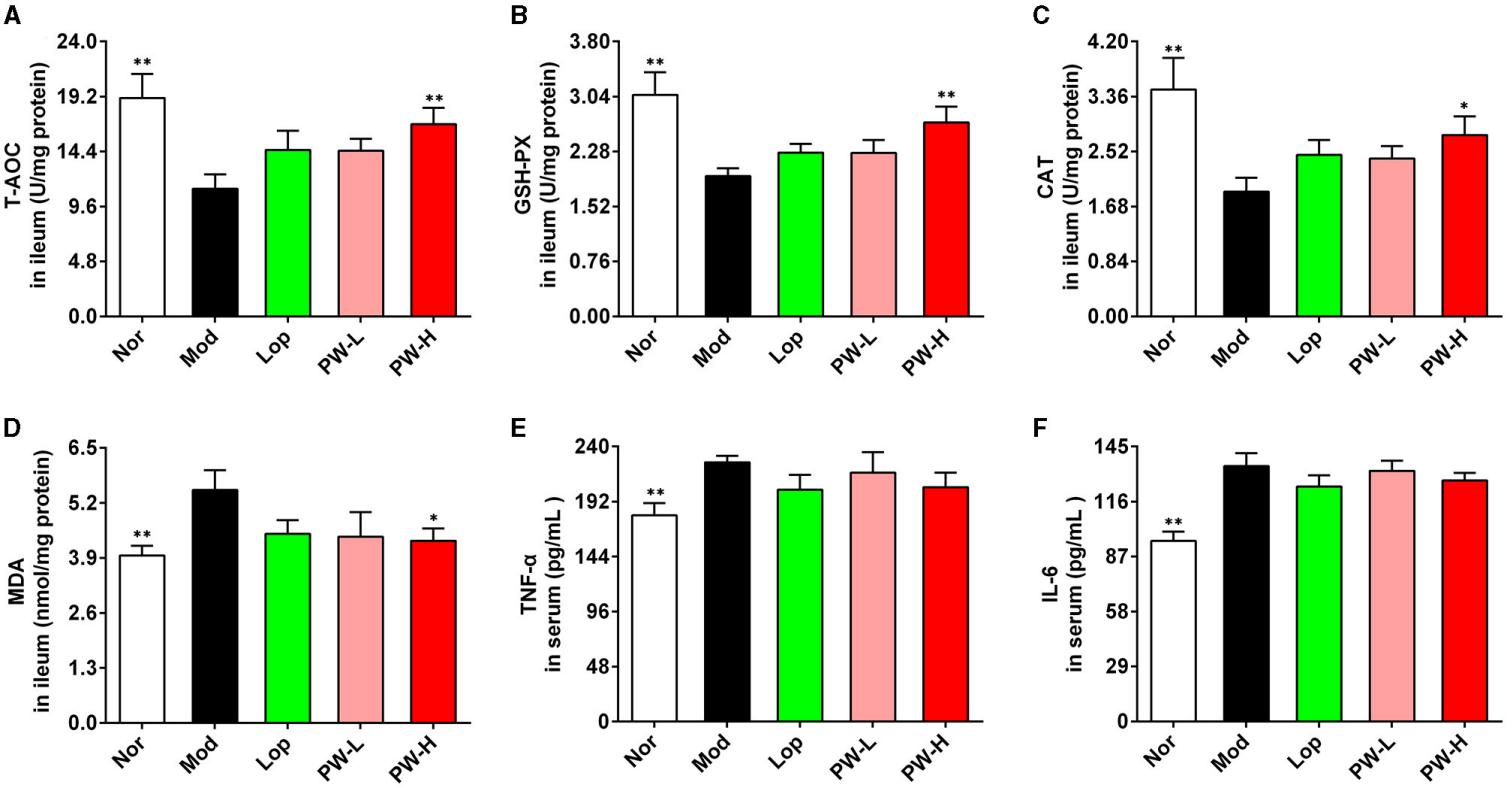
Effect of PW on oxidative damage of small intestine in mice with castor oil-induced diarrhea (*n* = 10, means ± SEM). **(A)** The activities of T-AOC in the ileum. **(B)** The activities of GSH-PX in the ileum. **(C)** The activities of CAT in the ileum. **(D)** The levels of MDA in ileum. **(E)** The levels of TNF-α in serum. **(F)** The levels of IL-6 in serum. *P < 0.05; **P < 0.01 vs. the Mod group.

Oxidative stress and inflammatory reactions play an important role in the occurrence and development of diarrhea, so the biochemical indexes related to these two aspects were determined. As demonstrated in Figures 3A–F, the activities of T-AOC, GSH-PX, and CAT in the ileum of mice in the Mod group were lower. The ileum MDA levels were higher, and the levels of TNF-α and IL-6 in serum were higher than those mice in the Nor group, respectively. Mice in the PW-H group had dramatically higher ileum T-AOC, GSH-PX, CAT activities, and lower ileum MDA levels than those in the Mod group. Lop and PW-L treatments tended to improvethese indexes. Moreover, Lop and PW did not affect the contents of TNF-α and IL-6 in the serum of the diarrhea mice. Overall, these results suggest that PW improved ileum oxidative stress damage in diarrhea mice without affecting the inflammatory response.

### 3.6 Effect of PW on α diversity and β diversity in mice with castor oil-induced diarrhea

**Figure 4:**
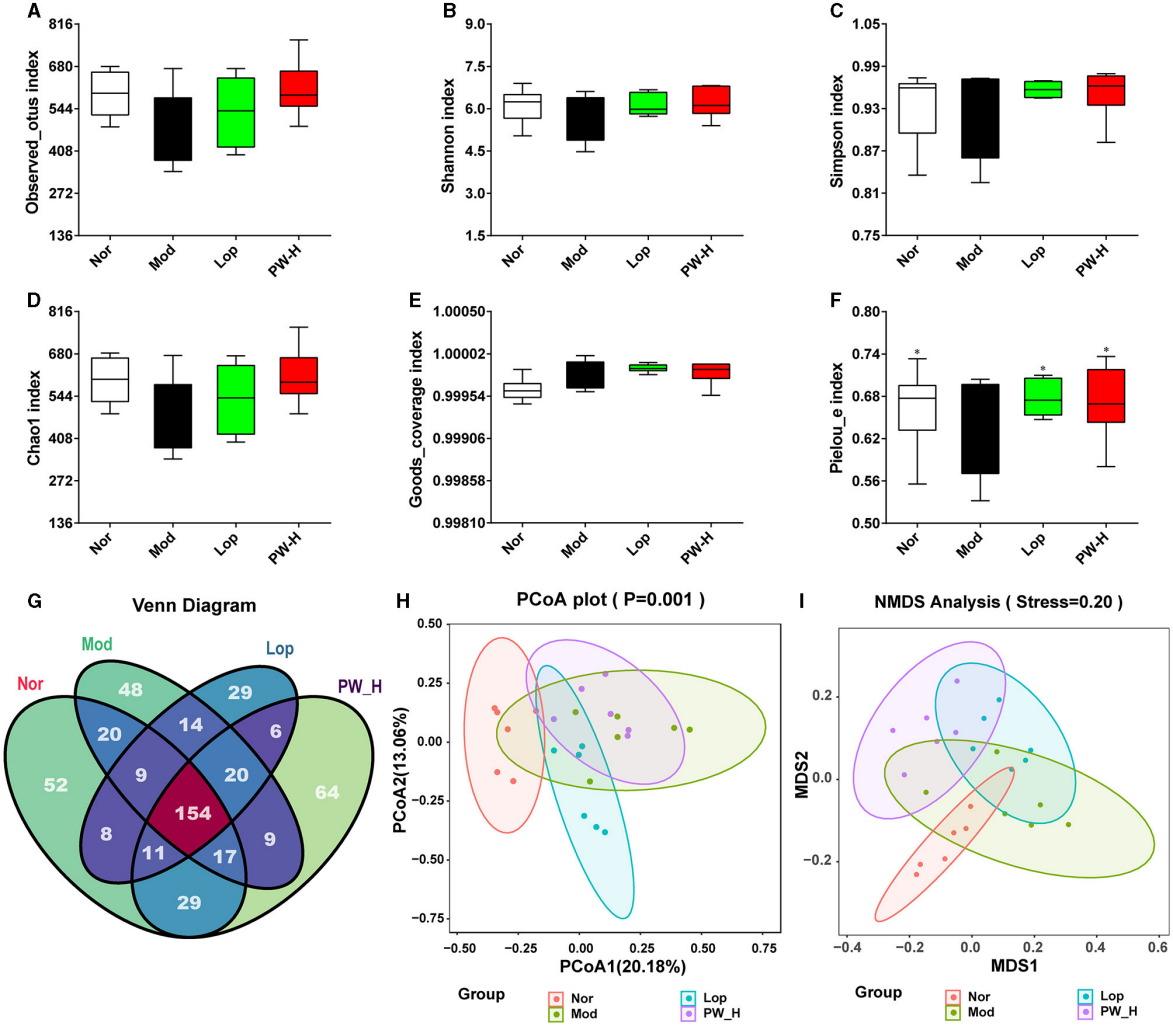
Effect of PW on α diversity and β diversity in mice with castor oil-induced diarrhea (*n* = 6, means ± SEM). **(A)** Observed_otus index. **(B)** Shannon index. **(C)** Simpson index. **(D)** Chao1 index. **(E)** Goods_coverage index. **(F)** Pielou-e index. **(G)** Venn diagram. **(H)** PCoA analysis diagram. **(I)** NMDS analysis diagram. *P < 0.05; **P < 0.01 vs. the Mod group.

To evaluate the effect of PW on the intestinal flora, we first assessed the α diversity and β diversity of intestinal flora in the feces of mice in each group. The results of α diversity are shown in Figures 4A–F. Compared to the Nor group, the index of observed_otus, Shannon, Simpson, and Chao1 in Mod group mice showed a downward trend, and the Goods_coverage index in the Mod group showed an upward trend. There was no statistical difference, but the Pielou-e index in the Mod group markedly decreased. Conversely, mice administered with Lop or PW showed improvements in these indexes compared to the Mod group, with a significant increase in Pielou's index. The Venn diagram of Figure 4G displayed 300, 291, 251, and 310 OTUs in groups Nor, Mod, Lop, and PW-H, respectively, with 154 reciprocal OTUs shared by all groups.

The results of β diversity are shown in Figures 4H, I, PCA and NMDS revealed that the gut microbiota of the Nor and Mod groups were significantly different and formed distinct clusters. However, after 15 days of Lop or PW administration, the results of PCoA and NMDS showed that the community structure in these groups was similar to that of the Nor group. Overall, these results suggest that PW supplementation can improve the structure and diversity of intestinal flora in the feces of diarrhea mice.

### 3.7 Effect of PW on the composition of intestinal flora in mice with castor oil-induced diarrhea at the phylum level

**Figure 5:**
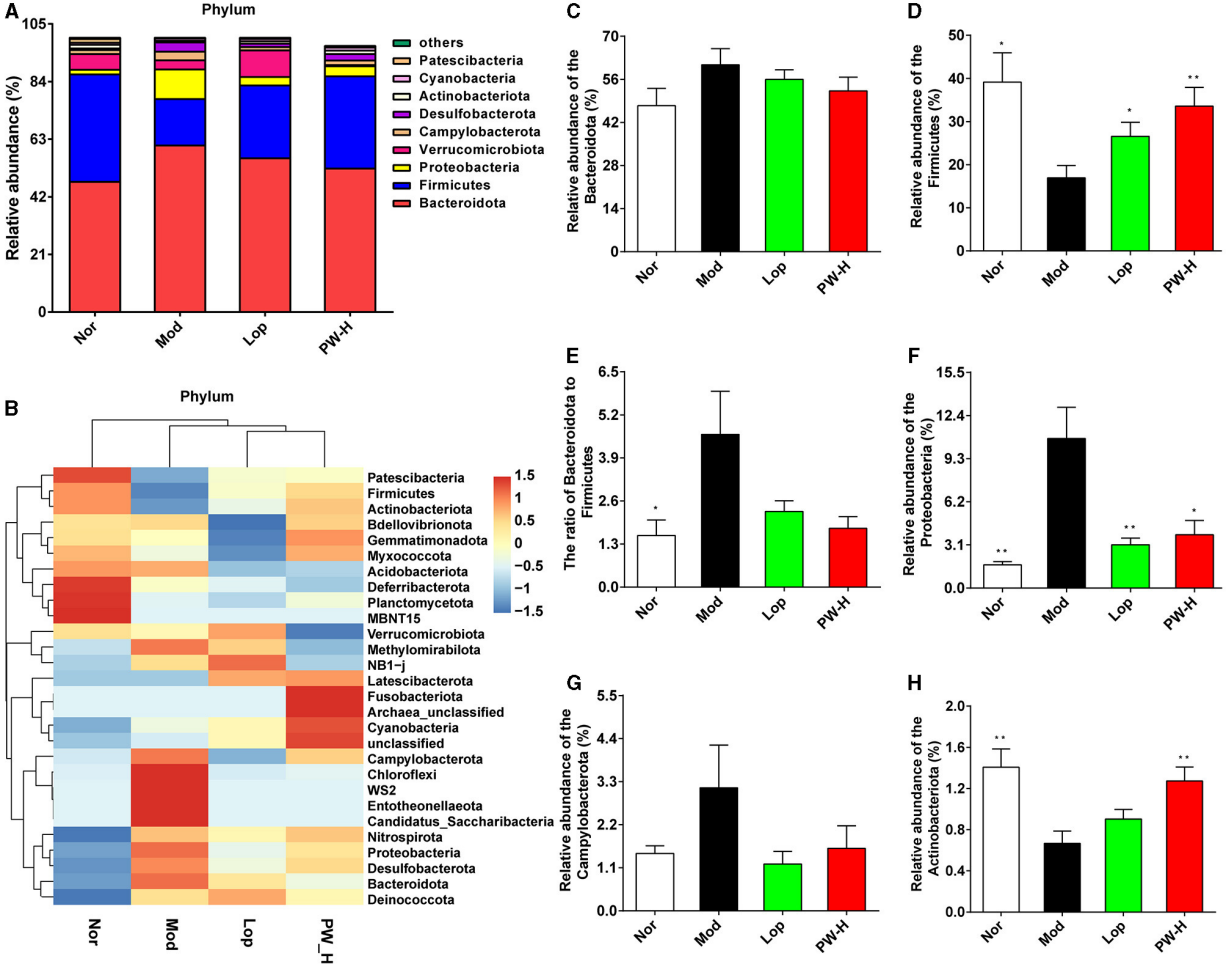
Effect of PW on the composition of intestinal flora in mice with castor oil-induced diarrhea at the phylum level (*n* = 6, means ± SEM). **(A)** Relative abundances of species at the phylum level. **(B)** Heat map of species distribution at the phylum level. **(C)** The relative abundance of Bacteroidota. **(D)** The relative abundance of Firmicutes. **(E)** The ratio of Bacteroidota to Firmicutes. **(F)** The relative abundance of Proteobacteria. **(G)** The relative abundance of Campylobacterota. **(H)** The relative abundance of Actinobacteria. *P < 0.05; **P < 0.01 vs. the Mod group.

To further analyze the influence of PW on the composition of intestinal flora, we examined the intestinal flora at the phylum level. As shown in Figure 5A, at the phylum level, the intestinal flora in the feces of mice in each group mainly comprises Bacteroidota, Firmicutes, Proteobacteria, and Verrucomicrobiota, accounting for about 85%. The distribution heat map in Figure 5B further illustrated that the microbial composition differed significantly between the groups, which was consistent with the columnar stacking diagram results. Additionally, we analyzed specific flora with significant differences, and the results are shown in Figures 5C–H. The relative abundance of Bacteroidota, Protecbacteria, Campylobacterota, and the ratio of Bacteroidota to Firmicutes in the Mod group were higher than those in the Nor group, and the relative abundance of Firmicutes and Actinobacteria in the Mod group were lower than those in the Nor group. However, after 15 days of Lop or PW treatment, the composition of intestinal flora in diarrhea mice was significantly improved and approached that of the Nor group. These findings collectively reveal that PW regulates the abundance of the intestinal microbial community and maintains intestinal homeostasis in diarrhea mice at the phylum level.

### 3.8 Effect of PW on the composition of intestinal flora in mice with castor oil-induced diarrhea at the genus level

**Figure 6:**
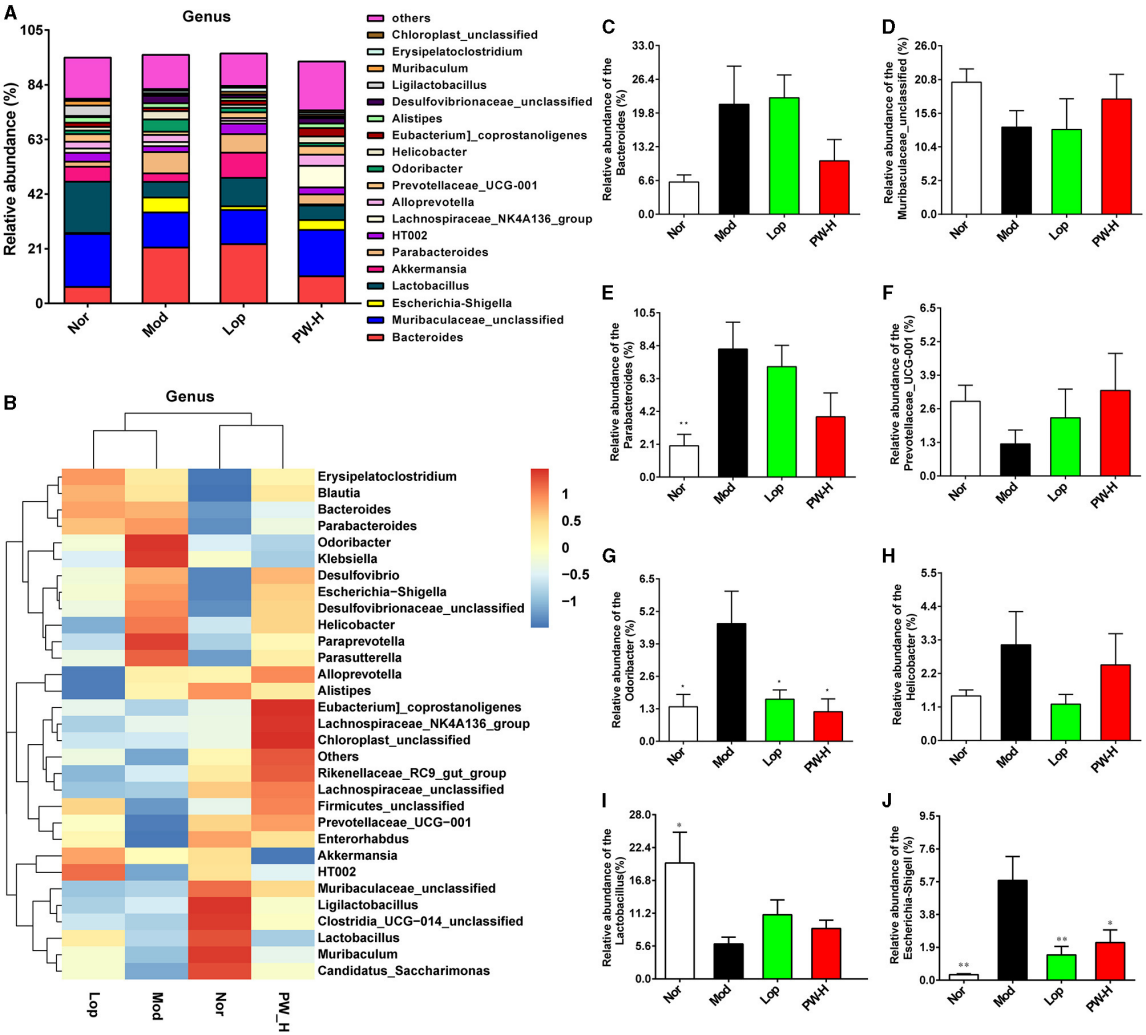
Effect of PW on the composition of intestinal flora in mice with castor oil-induced diarrhea at the genus level (*n* = 6, means ± SEM). **(A)** Relative abundances of species at the genus level. **(B)** Heat map of species distribution at the genus level. **(C)** The relative abundance of Bacteroides. **(D)** The relative abundance of Muribaculaceae-unclassified. **(E)** The ratio of Parabacteroides. **(F)** The relative abundance of Prevotellaceae-UCG-001. **(G)** The relative abundance of Odoribacter. **(H)** The relative abundance of Helicobacter. **(I)** The relative abundance of Lactobacillus. **(J)** The relative abundance of Escherichia-shigella. *P < 0.05; **P < 0.01 vs. the Mod group.

Then, the composition of intestinal flora in each group was analyzed at the genus level. As demonstrated in Figure 6A, at the genus level, the intestinal flora in the feces of the mice in each group is mainly composed of Bacteroides, Muribaculaceae-unclassified, Escherichia-shigella, Lactobacillus, Akkermansia, Parabacteroides and HT002, accounting for about 65%. The distribution heat map in Figure 6B indicated that the balance of intestinal flora in diarrhea mice was disrupted, and the microbial composition of each group differed significantly. However, Lop and PW could regulate these abnormal changes. Additionally, we analyzed specific genera with significant differences, and the results are shown in Figures 6C–J. Relative to the Nor group, the mice in the Mod group represented a higher relative abundance of Bacteroides, Parabacteroides, Odoribacter, Helicobacter, and Escherichia-shigella, as well as exhibited a lower relative abundance of Muribaculaceae-unclassified, Prevotellaceae-UCG-001 and Lactobacillus. After 15 days of Lop or PW intervention, these abnormal changes in community composition were significantly improved. Overall, these results suggest that PW can enhance intestinal flora's stability and mitigate castor oil's damage to intestinal flora at the genus level.

### 3.9 Effect of PW on the differentially abundant taxa in mice with castor oil-induced diarrhea

**Figure 7:**
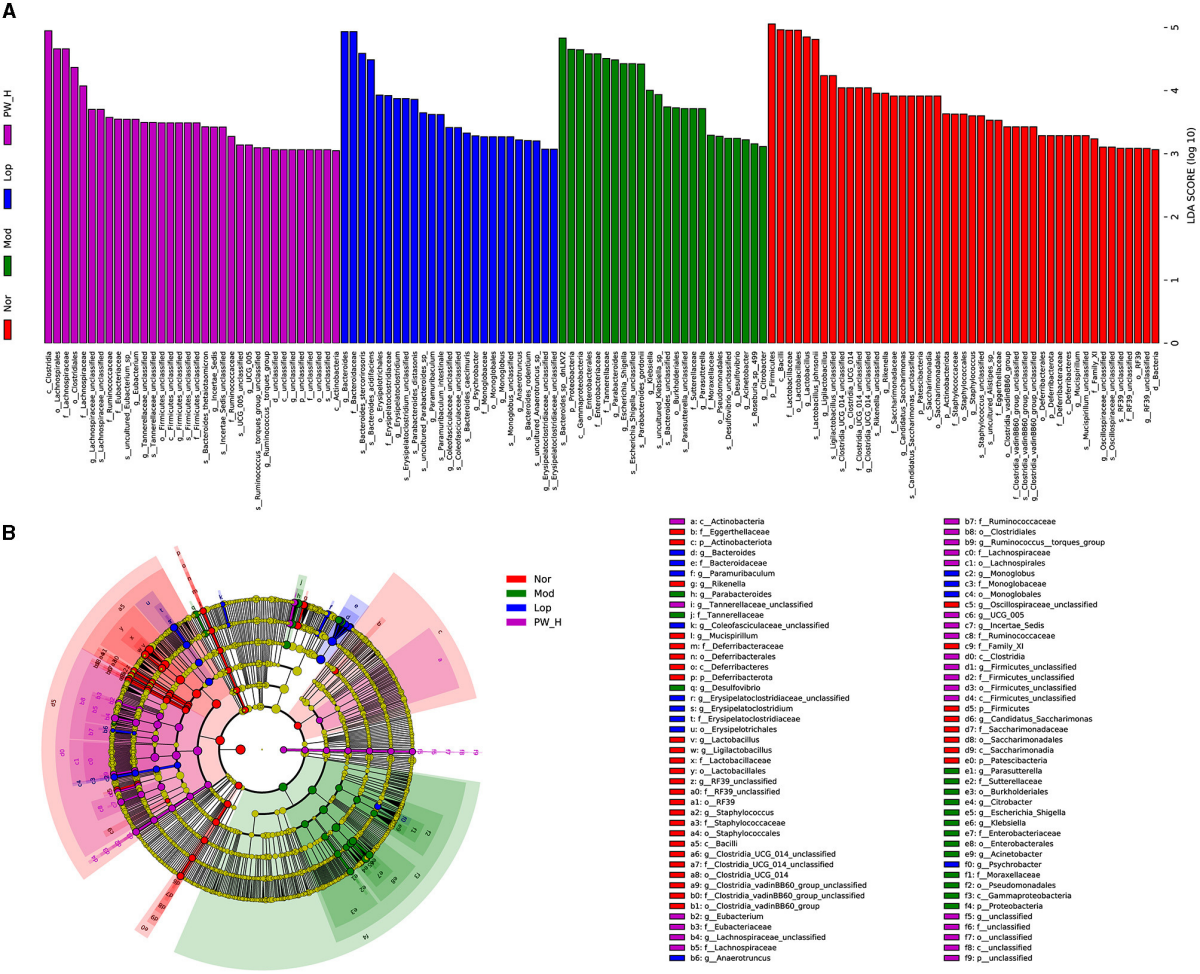
Effect of PW on the differentially abundant taxa in mice with castor oil-induced diarrhea (*n* = 6, means ± SEM). **(A)** Histogram of LDA values (LDA > 3) and **(B)** taxonomic cladogram obtained by LEfSe.

We used the linear discriminant analysis effect size (LEfSe) method to identify specific bacterial taxa. As shown in Figures 7A, B, based on a criterion of linear discriminant analysis score > 3, a total of 128 different OTUs were identified across the four groups. There are 45 biomarkers in Nor groups, and the dominant flora in feces were Firmicutes (phylum level), Bacilli (class level), Lactobacillales (family level), Lactobacillus (genus level), and Lactobacillus-Johnsonii (species level). There are 24 biomarkers in Mod groups, and the dominant flora in feces were Bacteroides-sp-dnLKV2 (species level), Proteobacteria (phylum level), and Gammaproteobacteria (class level). There are 25 biomarkers in Lop groups, and the dominant flora in feces were Bacteroides (genus level), Bacteroidaceae (family level), Bacteroides-stercorirosoris (species level), and Bacteroides-acidifaciens (species level). There are 34 biomarkers in PW-H groups, and the dominant flora in feces were Clostridia (class level), Lachnospirales (order level), Lachnospiraceae (family level), Clostridiales (order level), and Lachnospiraceae (family level). The above results revealed that PW may change the composition of intestinal microflora in mice with castor oil-induced diarrhea.

### 3.10 Correlation between the intestinal microbiota and diarrhea-related index

**Figure 8:**
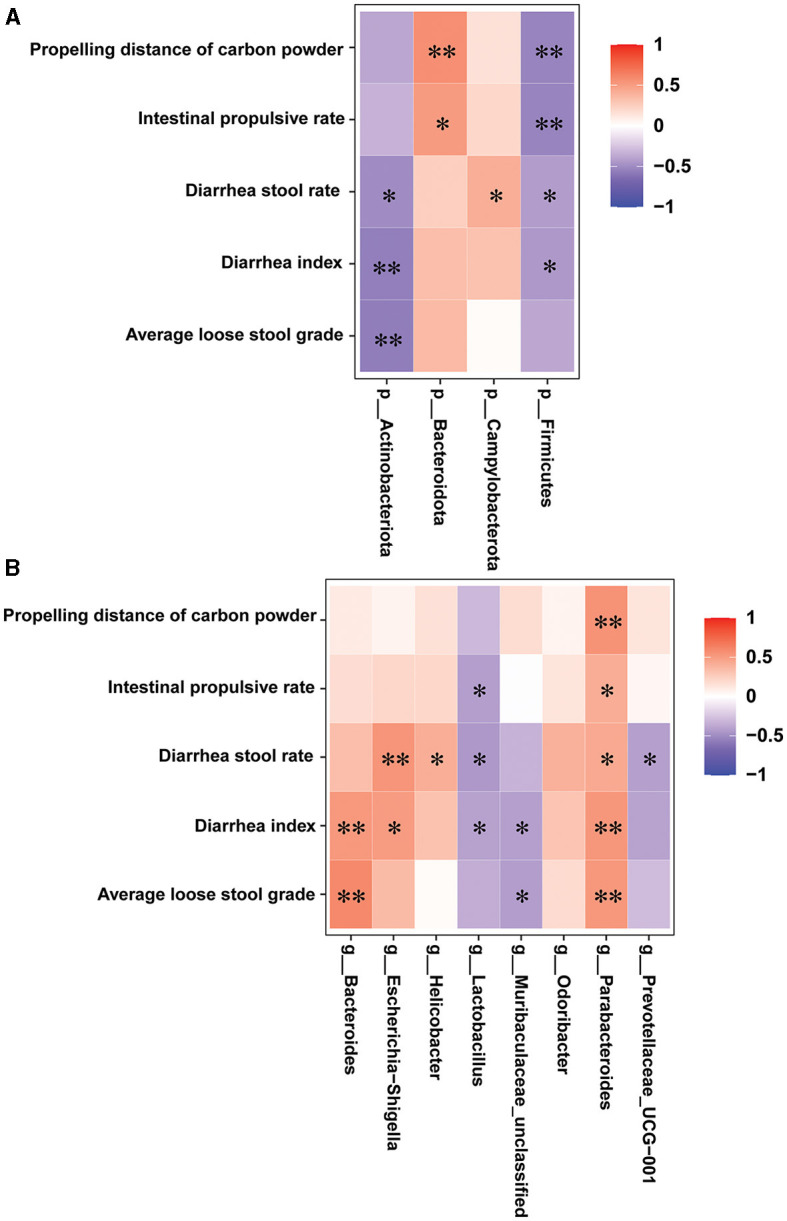
Correlation between the intestinal microbiota and diarrhea-related index (*n* = 6, means ± SEM). **(A)** Spearman correlation analysis between intestinal flora and diarrhea-related index at the phylum level. **(B)** Spearman correlation analysis between intestinal flora and diarrhea-related index at the genus level.

Spearman's correlation analysis was conducted to investigate the correlations between the diarrhea-related index and the gut microbes in all the groups. As illustrated in Figure 8, at the phylum level, the diarrhea stool rate, diarrhea index, and average loose stool grade were markedly and negatively associated with Actinobacteriota; the propelling distance of carbon powder and intestinal propulsive rate was markedly and positively associated with Bacteroidota; the diarrhea stool rate was markedly and positively associated with Campylobacterota; and the propelling distance of carbon powder, intestinal propulsive rate, diarrhea stool rate and diarrhea index were markedly and negatively associated with Firmicutes. At the genus level, the diarrhea stool rate, diarrhea index, and average loose stool grade were markedly and positively associated with Bacteroides, Escherichia-shigella, and Helicobacter; the intestinal propulsive rate, diarrhea stool rate, diarrhea index and average loose stool grade were markedly and negatively associated with Lactobacillus, Muribaculaceae-unclassified and Prevotellaceae-UCG-001; all diarrhea indicators were markedly and positively associated with Parabacteroides. Overall, the identified differential bacteria are closely related to the indicators for evaluating diarrhea.

## 4 Discussion

Diarrhea is a common gastrointestinal condition, particularly affecting children under 5 years old. It has been reported that more than 500,000 children die from severe diarrhea each year worldwide (20). Diarrhea is a leading cause of malnutrition and mortality in children, raising significant global concern (21). Pickled vegetables are a popular and well-known food in China. Although some studies suggest that pickled vegetables may alleviate diarrhea in children, no systematic research has been conducted. Thus, we investigated the effect of pickled vegetables on diarrhea and their mechanism in ICR mice with castor oil-induced diarrhea. Our study yielded the following results: (i) Pretreatment with PW significantly improved castor oil-induced diarrhea in ICR mice; (ii) PW enhanced the intestinal integrity of the ileum in diarrhea mice; (iii) PW ameliorated oxidative stress damage in the ileum of diarrhea mice; (iv) PW improved the structure and diversity of intestinal flora and modulated the fecal composition of intestinal flora in diarrhea mice. Therefore, PW may be a promising strategy for treating diarrhea.

Castor oil-induced diarrhea is a reliable and reproducible animal model for inducing acute diarrhea in experimental studies (22, 23). Castor oil can induce diarrhea for the following reasons: Castor oil induces diarrhea by stimulating intestinal mucosal cells and reducing the active absorption of Na^+^ and K^+^, leading to intestinal inflammation and diarrhea (24). Upon oral administration, castor oil releases ricinoleic acid through the action of intestinal lipase, which damages the intestinal mucosal barrier and alters mucosal liquid and electrolyte transport, resulting in diarrhea (25). Moreover, ricinoleic acid also promotes intestinal motility and causes diarrhea by binding to EP3 prostaglandin receptors and stimulating nerve reflexes (26); castor oil can stimulate intestinal peristalsis by promoting the release of histamine and tachykinin through binding to receptors on intestinal smooth muscle cells (26). In this experiment, mice administered castor oil exhibited higher rates of loose stool, average loose stool grade, diarrhea index, and intestinal propulsive rate compared to the Nor group. Moreover, in the Mod group, the intestinal mucosa showed swelling, villous detachment, disrupted normal small intestine structure, edematous epithelial cells, and defective intercellular junction structures. However, pretreatment with PW protected mice from castor oil-induced diarrhea and significantly reduced morphological damage to the ileum in diarrhea mice. These results suggest that PW has antidiarrheal effects and may be a promising candidate for treating castor oil-induced diarrhea.

The mechanism of diarrhea is complex and multifactorial, ultimately impacting gastrointestinal motility (27). Under normal conditions, tight junction proteins and intestinal epithelial cells form the intestinal epithelial barrier, which is crucial for maintaining gastrointestinal homeostasis and normal physiological function (28). Disruption of tight junctions and damage to the epithelial barrier compromise the polarity of intestinal epithelial cells, leading to leakage and diarrhea (29). ZO-1, along with other tight junction proteins, is essential for maintaining the integrity and stability of these junctions (30). The occludin protein plays a vital role in sealing intercellular junctions and maintaining epithelial cell permeability, structural integrity, and barrier function (31). Oxidative stress is a major cause of intestinal barrier dysfunction and various gastrointestinal diseases. In diarrhea patients, persistent inflammation and barrier damage lead to excessive free radical production and oxidative stress, which causes oxidative damage to epithelial cells and tissues (32).

Additionally, oxidative stress exacerbates inflammation, affecting the morphology of the intestinal mucosa and its absorption function, resulting in diarrhea (33). Castor oil has been shown to promote diarrhea by inducing oxidative stress in the gastrointestinal mucosa. Our results indicated that PW increased the expression levels of ZO-1 and occludin proteins, reversed the decrease in T-AOC, GSH-PX, and CAT activities, and reduced MDA levels. Consequently, the protective effects of PW on castor oil-induced diarrhea in mice may be related to enhanced intestinal integrity and reduced oxidative stress.

The intestinal flora, a vast micro-ecosystem within the human body, is closely linked to health (34). An imbalance in intestinal flora can lead to various digestive system diseases, such as acute and chronic diarrhea and gastroenteritis. Patients often show an imbalance in dominant intestinal flora (e.g., Enterococcus, Bifidobacterium, Lactobacillus, and Bacteroides) with a reduction in resident flora (9). The intestinal microflora plays a crucial role in host metabolism, systemic immunity, and neurobehavioral characteristics (35). Recent research has highlighted that intestinal microecological imbalances, induced by water and electrolyte metabolism disturbances, are significant causes of diarrhea (9, 11). When foreign bacteria invade the intestine, they disrupt the original diversity and abundance of intestinal microorganisms, affecting intestinal homeostasis, particularly in diarrhea patients. Intestinal microflora has a protective effect against diarrhea in sterile and antibiotic-treated mice (36). Microbial interventions can prevent and improve diarrhea by regulating the composition of intestinal flora (37). In this study, the composition of the microbial community in the feces of the Mod group was significantly different from that of the Nor group. Supplementation with PW reversed the abnormal diversity of intestinal microflora induced by castor oil, as evidenced by changes in α diversity and β diversity in the feces of diarrhea mice. We further analyzed the intestinal flora at the phylum and genus levels. Firmicutes and Bacteroides are key phyla regulating host inflammation and adaptive immunity and are closely related to many diseases (38). Firmicutes typically account for a high proportion of the intestinal microflora of healthy individuals, decreasing with disease progression (39). Excessive growth of Firmicutes can produce metabolic endotoxins, such as lipopolysaccharides, leading to systemic inflammation (40). The Bacteroides/Firmicutes (B/F) ratio reflects the impact of intestinal flora on health, with a decreased ratio indicating inflammation and immune imbalance (41). In this study, however, we observed an increase in the B/F ratio with diarrhea. This discrepancy may be due to varying environmental influences, such as diet and species differences between humans and mice. Additionally, the proliferation of Proteobacteria has been identified as a microbial marker for the progression of diarrhea (42). Campylobacterota is a common pathogen in foodborne gastroenteritis, while Actinobacteriota can use different carbohydrates and decompose many organic compounds (43, 44). Interestingly, our study showed that PW tended to reverse these abnormal changes. Moreover, supplementation with PW also affected other communities in the genus, such as Bacteroides, Muribaculaceae-unclassified, Parabacteroides, Prevotellaceae-UCG-00, Odoribacter, Helicobacter, and Lactobacillus Escherichia-shigella.

Moreover, the results of the correlation between the intestinal microbiota and diarrhea-related index further indicated that the identified differential bacteria are closely related to the indicators for evaluating diarrhea. These results suggest that the improvement of castor oil-induced diarrhea by PW may be related to its ability to remodel the composition and structure of intestinal microflora. The above results show that PW could improve castor oil-induced diarrhea in ICR mice, but it is not clear which components are acting. The main reasons for not conducting component analysis are as follows: (i) There are few reports about whether PW can improve diarrhea. This study aims to clarify the effect of PW on diarrhea, which components are effective, and our future concerns. (ii) A large number of microorganisms, amino acids, vitamins, and other substances are produced in the production process of pickles (45). The anti-diarrhea effect of PW may be the result of the joint action of many substances, and a single component may have no effect. (iii) The composition, structure, and quantity of nutrients and bacteria in PW are influenced by many factors, such as geographical and climatic conditions, fermentation temperature and time, production procedures, and vegetable types (46, 47). (iv) In addition to conventional substances, studies have shown that Sichuan pickle water contains more microorganisms, such as *Enterococcus faecalis, Lactobacillus delbrueckii* subsp. lactis, *Leuconostoc mesenteroides, Lactiplantibacillus plantarum* subsp. plantarum, *Lacticaseibacillus casei*, and *Lacticaseibacillus zeae* (45, 48–50). Nevertheless, it will be more convincing and meaningful if we can analyze the components of kimchi water and clarify which substances play a role in anti-diarrhea, which is also a direction worthy of further study.

However, this study had several limitations. First, we did not capture images of the fecal characteristics or assess the mental state of the mice during the experiment. Additionally, while we investigated the antidiarrheal effects of PW, we did not analyze its specific components, leaving it unclear which ones contribute to its efficacy. Second, although we observed that PW mitigates oxidative stress-induced damage in the small intestine and inhibits inflammatory reactions in the serum of diarrhea-induced mice, the underlying molecular mechanisms were not thoroughly explored. Further research is needed to investigate these mechanisms to provide a more robust and comprehensive understanding of PW's effects. Finally, this study was conducted in mice, and the findings have been verified in clinical trials. Therefore, further studies are required to explore the potential of PW in treating diarrhea in humans.

**Figure 9:**
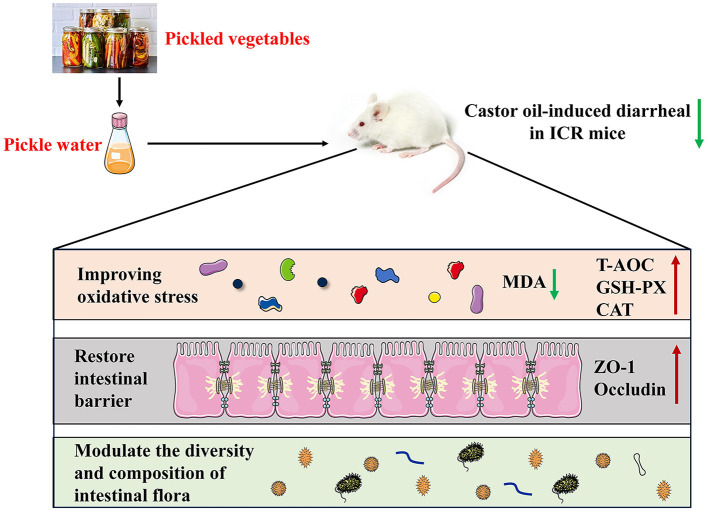
A proposed schematic diagram for the protective mechanisms of PW against castor oil-induced diarrhea in ICR mice.

In conclusion, our findings demonstrated that PW acts as a protective agent against castor oil-induced diarrhea in ICR mice. The potential mechanisms underlying the antidiarrheal effects of PW may involve mitigating oxidative stress, restoring tight junctions between intestinal mucosal cells, and regulating gut microbiota (Figure 9). These findings highlight the therapeutic potential of PW in treating diarrhea and provide innovative insights into potential treatment strategies.

## Data Availability

The original contributions presented in the study are included in the article/supplementary material, further inquiries can be directed to the corresponding author.
